# Therapeutic Modulation of the Complement Cascade in Stroke

**DOI:** 10.3389/fimmu.2019.01723

**Published:** 2019-07-30

**Authors:** Alison R. Clarke, Brandon R. Christophe, Anadjeet Khahera, Justin L. Sim, E. Sander Connolly

**Affiliations:** Cerebrovascular Research Laboratory, Department of Neurological Surgery, Columbia University Irving Medical Center, New York, NY, United States

**Keywords:** complement, vascular disorders, complement activation, cerebral blood flow, complement cascade, stroke therapy

## Abstract

Stroke is a leading cause of death and disability worldwide and an increasing number of ischemic stroke patients are undergoing pharmacological and mechanical reperfusion. Both human and experimental models of reperfused ischemic stroke have implicated the complement cascade in secondary tissue injury. Most data point to the lectin and alternative pathways as key to activation, and C3a and C5a binding of their receptors as critical effectors of injury. During periods of thrombolysis use to treat stroke, acute experimental complement cascade blockade has been found to rescue tissue and improves functional outcome. Blockade of the complement cascade during the period of tissue reorganization, repair, and recovery is by contrast not helpful and in fact is likely to be deleterious with emerging data suggesting downstream upregulation of the cascade might even facilitate recovery. Successful clinical translation will require the right clinical setting and pharmacologic strategies that are capable of targeting the key effectors early while not inhibiting delayed repair. Early reports in a variety of disease states suggest that such pharmacologic strategies appear to have a favorable risk profile and offer substantial hope for patients.

## Public Health Impact of Stroke

Worldwide, stroke is the second leading cause of death, and the third leading cause of disability-adjusted life-years (DALYs), with a staggering lifetime risk after age 25 of 26.5% (1, 2). Even more concerning is the fact that the global incidence for both major stroke subtypes is increasing (37% for ischemic and 47% for hemorrhagic over the last 20 years). Moreover, despite broad advances in general medical care, stroke-related deaths and DALYs have also increased by 26 and 19%, respectively. It is also important to note that stroke is not merely a disease of the old and wealthy, as those living in low- and middle-income countries and those younger than 75 years of age make up the majority of victims.

## Current Treatment of Acute Ischemic Stroke

Since 1995, there have been a number of major advances in the prevention and treatment of stroke, most notably, the widespread advent of contemporary anti-platelet therapy, statin therapy, and the introduction of highly effective anti-hypertensive regimes (3). Particularly impactful has been the development of intravenous pharmacologic thrombolysis and intra-arterial mechanical thrombectomy following acute ischemic stroke (4). That said between 25 and 50% of patients who are eligible for intravenous therapy within 9 h of stroke onset still suffer at least moderate disability (5–7). For those presenting with anterior circulation large vessel occlusions, substantial reperfusion is now achievable for 85% of patients, in some instances as late as 24 h after onset. Yet, 30–50% of patients eligible for this therapy are disabled as well, despite fairly small areas of core infarction (8, 9). Together, these data suggest that therapeutic targeting of post-reflow microvascular failure may have significant clinical utility for a substantial proportion of ischemic stroke patients.

## Importance of Inflammatory Microvascular Failure on Stroke Outcome

While post-stroke inflammatory microvascular failure is governed by multiple biological cascades (10), our group long ago implicated the complement cascade as one of the central players that is potentially druggable (11). The first data suggesting that the complement cascade was involved in secondary tissue injury following re-perfused stroke dates to the late 1990s when we showed that soluble complement receptor 1 (sCR1), and its Sialyl-Lewis X glycosylated form (sCR1-sLex), which targeted complement inhibition to sites of activated endothelium, reduced ischemic tissue injury, improved outcome and penumbral blood flow (11). Nevertheless, 20 years of research has yet to result in the development of a clinically relevant anti-complement or even anti-inflammatory therapy for re-perfused stroke. Below we review the existing data implicating the complement cascade in cerebral ischemia reperfusion injury. We will not attempt to reiterate all that has been presented in two recent and exhaustive reviews (12, 13), but rather will attempt to present the data in a manner that facilitates clinical translation. In doing so, we will attempt to address the apparent inconsistencies in the data obtained from different model systems, as well as, the differences in the effect of acute and subacute treatment on cell death, cell survival and tissue regeneration.

## *In Vitro* Data Examining the Role of Complement in Cerebral Tissue Homeostasis and Ischemic Injury

Primary astrocyte, neuronal, and glial cell cultures express many of the complement cascade components (14). Together with resident microglia, these cells generally utilize the complement cascade to respond to infection. In addition, the complement cascade appears to be involved in both central nervous system development and tissue repair, in part by directing synaptic pruning and plasticity (15). Specifically, C1q and C3 tag weaker synapses for microglial removal and C3a and C5a and their receptors appear to be involved in cerebellar development (16, 17). C3a has also been shown to be involved in neuronal differentiation and maturation of neural progenitor cells (NPCs). It additionally guides migration of NPCs using an ERK 1/2 signaling pathway (18) and protects astrocytes from ischemia-induced cell death (19). C5a has also been shown to make microglia more resistant during ischemia by limiting the toxic effects of glutamate (20). Both C3a and C5a appear to be protective in culture against NMDA- and Kainate-induced neurotoxicity, respectively (21, 22). C1q drives neuronal survival in primary cultures and sublytic MAC has a similarly positive effect on oligodendrocyte progenitor cell cultures (23, 24). Complement receptor 2 (CR2) via C3d binding may regulate hippocampal neurogenesis in the adult rodent dentate nucleus (25).

Simulated ischemia via oxygen glucose deprivation (OGD) induces the production of neuronal C1q and C3 which are associated with pro-apoptotic caspase-3 activation (26). Inhibiting C3 expression via siRNA enhances cultured neuronal viability (27). Blocking C5a reduces ischemia-induced apoptosis in culture (28). These results were less dramatic in human neuronal cultures due to the high expression of complement modifying inhibitors CD59, CD46, and CD55 (29).

Together the *in vitro* data suggests that in the absence of ischemic stress, components of the complement cascade are for the most part important to cell survival and appear to be potentially helpful in recovery of function after injury. By contrast, following ischemic stress, complement cascade activation appears to mediate apoptotic cell death, at least acutely.

## *In Vivo* Experimental Data Examining the Role of Complement in Cerebral Tissue Injury and Repair Following Ischemic Injury

### Initiation of Complement Activation Following Cerebral Ischemia Reperfusion

It appears that ischemia-reperfusion results in the upregulation of the complement cascade in a number of ways. Recently, some data suggests that plasmin and thrombin released at the site of thromboembolism may play a role in complement activation independent of the classical, alternative, and lectin pathways (30–32). That said, more recent data in non-human primates suggests that this process is unlikely to be the source of most of the complement generated following cerebral ischemia-reperfusion injury in the context of most reperfused human ischemic strokes (33). Therapeutic treatment with recombinant tissue plasminogen activator also is known to activate the complement cascade via a plasmin-mediated MBL-independent extrinsic/alternative pathway, but the degree to which this is relevant in clinical stroke is similarly unknown (34).

What is clear, however, is that, even in the absence of physiologic or therapeutic clot lysis, the ischemic endothelium expresses neo-epitopes, such as non-muscle myosin, annexin IV, and a subset of phospholipids that are recognized by circulating self-reactive IgM antibodies (35). Following ischemic injury, mannose-binding lectins (MBL) deposit on the activated endothelium. The antibody complexes activate the lectin pathway by reacting with MBL and the ficolins. Carbohydrate-bound MBL then cleaves C2 and C4, forming the C4b2a (C3-convertase), which cleaves C3, and initiates the distal complement cascade (36). Preventing MBL function through genetic deletion or pharmacological manipulation is thought to be protective (37). Furthermore, even in the absence of thrombin or plasmin-mediated activation of the alternative pathway, amplification of this process appears to be driven by the alternative pathway. Efforts to block either this alternative pathway activation (38) or neo-epitope dependent complement activation (39) have proven extraordinarily effective in improving outcome in rodent models of cerebral ischemia-reperfusion injury (cIRI), as evidenced by reductions in infarct size, reduced apoptotic cell death, reduced neurological deficit scores throughout the recovery period, reduced gliosis, improved neurogenesis and angiogenesis even when employed 24 h post-reperfusion, with the models including aged mice, female mice, and mice exposed to varying severities of ischemic injury (40).

Evidence that the classical pathway is not the primary initiator of complement cascade activation in focal cIRI, at least in adult animals, comes from the observation that C1q deficient mice (C1q^−/−^) are not protected from cIRI and the C1q that is initially expressed appears to be produced predominantly by microglia rather than neurons (37, 41, 42). By contrast, C1q/MBL^−/−^, MBL^−/−^, and factor H^−/−^ mice are all protected from focal cIRI, albeit to differing degrees depending on variations in the model system utilized (43, 44). Moreover, animals treated with a lectin pathway inhibitor, Polyman 2, or, as previously mentioned, an anti-MBL antibody are similarly protected as are those treated with CR2-fH, a targeted alternative pathway inhibitor (44, 45).

Despite the overwhelming amount of data implicating upstream initiation of the complement cascade by the alternative and lectin pathways, there are further data in an alternative model of cerebral ischemia that require mention. Neonatal mice subjected to a global ischemia reperfusion injury are protected by genetic deletion of C1q (46). In this model system, the severity of the ischemia is markedly less and the apoptotic burden is likely more significant than it is in focal stroke in adults (47). Given that this is dissimilar from what is seen in focal cIRI in adult animals including humans, and the fact that C1q deficiency may pre-dispose to epilepsy as a result of uncontrolled synaptogenesis, with post-stroke epilepsy worsening outcomes (48), attempts at therapeutic manipulation in clinical cIRI should likely avoid strategies which include acute C1q blockade. In fact, this hypothetical downside to early C1q blockade, may in part explain the failure of sCR1 treatment to improve cIRI in non-human primates (49).

### Downstream Mediators of Complement-Dependent Acute Tissue Injury Following Cerebral IRI

Cleavage of C3 and generation of the anaphylatoxin (C3a), as well as the opsonins (iC3b, C3dg and C3d) and formation of C5 convertase (from C3 and C3b), which in turn generates C5a and C5b which aggregate with C6, C7, C8 and several C9s to form the MAC, could all potentially be involved in tissue injury following re-perfused stroke. However, most of the data suggest that, early following reperfusion, tissue injury is mediated principally by C3a and to a lesser extent C5a while MAC is not pathologically important, but rather, simply, a marker of complement cascade activation (42, 50, 51). The first evidence of this was the finding that C3^−/−^ mice were markedly protected, while C5^−/−^ mice were not protected (38, 42). Similarly, C3aRA treatment early following stroke not only protected mice but enhanced delayed, post-stroke neurogenesis (50, 52). C6^−/−^ mice were also not protected (44), and while C5a receptor antagonist (C5aRA) (53) and C5 anti-body (54) treated mice were protected, the protection was more modest. These data together with the remarkably effective C3 site-directed therapy via CR2-Crry treatment (39), lead one to conclude that most complement mediated tissue injury following focal cIRI occurs as a result of C3 cleavage with the generation of C3a and binding of the C3aR. C5a binding of C5aR likely plays a lesser but collaborative role.

### Complement Cascade in Cerebral Repair and Recovery

While C3 depletion or blockade is protective in re-perfused stroke, especially when blockade is acute (52), germ-line depletion or chronic blockade results in loss of functional protection at later time points as stroke recovery is impaired by decreased neurogenesis. This impairment of neurogenesis by complement blockade was most pronounced in models of experimental rodent stroke lacking reperfusion where even acute blockade is known to be ineffective (55). In an equally non-reperfused model, intranasal treatment of mice undergoing permanent photothrombotic stroke with C3a in the late subacute period resulted in enhanced functional recovery (56). These data, together with the *in vitro* data presented above, strongly argue for limiting the clinical use of anti-complement therapeutics to the acute period during which reperfusion injury is most active (e.g., <72 h post-reperfusion). Table 1 summarizes the current knowledge of different components of the complement cascade and their roles in the central nervous system, tissue repair, ischemia reperfusion injury, and human stroke.

**Table 1:** The Role of the complement cascade in central nervous system cell clearance, viability, and cerebral tissue repair as well as in experimental cerebral ischemia reperfusion injury and human stroke.

**Component of the complement cascade**	**Significant role in cell clearance**	**Significant role in experimental inflammatory microvascular no-reflow**	**Significant role in cerebral tissue repair**	**Significant role in cerebral cellular viability *in vitro***	**Implicated in the pathogenesis of adult human stroke**
**C1q (classical pathway)**	Yes (15–16)	No (44–46)	Yes (27–9)	Yes (26)	No
**Mannose binding lectin (lectin pathway)**	–	Yes (39, 45, 47, 49)	–	–	Yes (70, 72, 74–6, 78)
**Factors D/B/H (alternative pathway)**	–	Yes (40, 41, 43, 48)	–	–	
**C3**	Yes (16)	Yes (40, 41, 45)	Yes (23)	Yes (30, 36, 74)	Yes (66, 69, 84)
**C3a/C3aR**	No	Yes (54, 56–7)	Yes (57, 64)	Yes (17, 21, 24)	Yes (67)
**C3b**	Yes	–	–	–	No
**CR1**	Yes	Yes (41)	–	–	No
**CR2**	–	–	Yes (28)	–	No
**C5**	–	Yes (63)	–	–	Yes (81)
**C5a/C5aR**	No	Yes (62)	–	Yes (22, 25, 31)	Yes (71)
**C5b−9**	Yes	No (48)	Maybe	Yes (27)	Yes (86)

## Clinical Data from Humans Suffering Stroke Suggesting that Modification of Complement Cascade Activation Might Improve Outcome

### Evidence of Complement Cascade Activation in Human Ischemic Stroke and Association With Outcome

Polymorphisms in C3, C5, and factor H are associated with an increased incidence of ischemic stroke as are higher plasma levels of C4 and C5 (57–60). Polymorphisms in MBL are associated with a better outcome after ischemic stroke (43, 61). In patients suffering a stroke, plasma levels of C3a, C3, C4, C5, C5a, factor B, MBL, MASP-1/2, and MAC are all elevated and ficolin-1, ficolin-2, and ficolin-3 reduced (62–66). Increased serum levels of C3, C3c, C4, and MBL are all associated with increased stroke severity, and patients who are MBL-sufficient have higher C3 plasma levels and suffer worse stroke outcomes (43, 67). Reduced early (within 6 h of onset) ficolin-1, a marker of activation of the lectin pathway, is independently associated with unfavorable outcome in adult human stroke (68). Moreover, postmortem studies have identified both complement and IgM deposition in the brain after stroke (69).

In addition, there is data in several other clinical scenarios of cerebral ischemia reperfusion injury where the complement cascade is activated. In cardiopulmonary bypass, glial injury is associated with complement cascade activation (70), and while anti-C5 monoclonal antibody therapy with a problematically long half-life did not improve overall cognition, it did improve outcomes in visuo-spatial functioning both acutely and at 1 month (71). Clinically relevant cerebral reperfusion injury is also experienced by a quarter of patients undergoing carotid endarterectomy for carotid stenosis (72). Interestingly MBL, C3, factor H, and C5 polymorphism correlate with both early and late cognitive dysfunction, and C3a levels are not only increased in those injured, but also correlate with duration of cross-clamp (a surrogate of severity of ischemia), and are predicted by MBL polymorphisms (73–75). In patients with carotid stenosis, the degree of plaque burden, the severity of the stenosis and the instability of the plaque is correlated with elevated circulating levels of MAC and reduced circulating levels of ficolin-2 (76, 77). Finally, in comatose post-cardiac arrest patients, who suffer global cerebral ischemia reperfusion, C3a/C3 ratios independently predict survival (78).

### Evidence of Complement Cascade Activation in Hemorrhagic Stroke and Association With Outcome

While less common than ischemic stroke, hemorrhagic stroke is associated with even worse outcomes and also exhibits complement cascade activation. This is important given that clinical efforts to revascularize ischemic stroke patients can result in either intracerebral or subarachnoid hemorrhage. In subarachnoid hemorrhage, C3a and M-ficolin levels are elevated in serum, cerebrospinal fluid and in the brain parenchyma and they are correlated with secondary ischemic injury and functional outcome (79–81). In patients suffering intracerebral hemorrhage, M-ficolin levels are similarly elevated and polymorphisms in factor H as well as iC3b levels are associated with functional outcome and mortality (51, 82). These observations support the experimental intracerebral hemorrhage data from rodents showing that inhibition of C3a and C5a receptors improve outcome with the effect at least in part due to amelioration of IL-1 dependent perihematomal edema formation (83, 84).

### Blood-Brain Barrier Dysruption Following Human Stroke and Its Implication for Systemic Intravascular Complement Blockade vs. Intracerebral Blockade

There has been considerable speculation regarding the importance of the blood brain barrier in developing therapeutic strategies for central nervous system disease. Cerebral ischemia reperfusion injury differs from most of these other diseases in two important aspects. First, the blood brain barrier is opened to some degree following cIRI (85–87). Moreover, this opening is most profound in the time frame where anti-complement strategies are likely to prove most protective (39) prior to when regenerative and repair mechanisms appear most important. Secondly, it appears that even if anti-complement strategies fail to cross the blood brain barrier, much, if not most, of their beneficial effect is likely to be mediated intravascularly at the level of the cerebral arteriole and capillary. In this respect, anti-complement therapies are distinct from most traditional neuroprotective strategies aimed specifically at increasing the tolerance of neurons to ischemia and the downstream intracellular cascades that result. That said, even anti-complement therapies with large molecular sizes, such as Eculizumab, appear to be able to cross even a moderately impaired blood brain barrier in neuromyelitis optica and improve clinical outcome (88). This portends even better results for smaller anti-complement therapies.

### Complement Blockade in the Setting of Intravenous Thrombolytic Therapy

The complement cascade has been implicated in cross-talk between the inflammatory cascade and the thrombotic cascade. While in some settings complement inhibition has been shown to prolong bleeding times, the use of a variety of anti-complement therapies together with intravenous thrombolytic therapies in cIRI has consistently shown enhanced outcomes with no increase in bleeding and in some instances a reduction in hemorrhagic conversion, a complication of ischemic stroke, possibly due to stabilization of the blood brain barrier (34, 89). This is additionally important for use with pharmacologic thrombolytic therapies that can exacerbate hemorrhage and edema (34).

### Clinically Available Complement Cascade Modifying Therapies for Clinical Translation

While it has widely been reported that anti-inflammatory strategies shown to be effective in experimental model systems fail to improve outcome following human stroke, most of this failure can be explained by the fact that these strategies are only useful in limiting reperfusion injury. Unfortunately, the patients included in these largely underpowered phase II trials do not have documented reperfusion and less than a third would even be expected to have a penumbra sufficient to demonstrate protection. Scientifically unjustified administration schedules based on incompletely studied pharmacokinetics has only further hindered efforts. Even so, despite all of these deficiencies, the most recent trials have shown some modest signal of benefit in both functional outcome and quality of life (90).

To date, anti-complement strategies have not been trialed in human stroke. Given the data presented above one would expect that either inhibition of the lectin and, or, alternative pathway might prove useful, as might inhibition of C3 convertase or the downstream anaphylatoxins, C3a and C5a. While recombinant C1-INH is clinically available as Cinryze, Ruconest, and Berinert, an important, but fairly rare side effect is pro-thrombotic events which would be potentially disastrous in the setting of ischemic stroke (91, 92). While anti-C5 Eculizumab, is well-studied and has proven beneficial for a variety of diseases, both in and outside of the central nervous system, its inability to block C3a binding of C3aR and its extremely long half-life (>10 d) makes its development less attractive. Selective alternative pathway inhibitors might also prove somewhat beneficial but the anti-C3 compstatin analogs seem to hold the most promise. Mechanistically, the latter block C3a and C5a generation, regardless of the upstream pathway responsible for activation of the cascade. They also appear to be safe in a variety of human diseases and have been designed with short half-lives that allow for ischemic protection without negatively impacting repair and recovery (93). The complement inhibitors currently in clinical development are listed in Table 2.

**Table 2:** Complement inhibitors in clinical development with potential utility in clinical stroke (94).

	**C1-INH berinert cinryze**	**OMS-721**	**ACH-4471**	**LPN-023**	**ACH-5228**	**Novartis oral agent**	**Small molecule anti-fD**	**APL-2/9**	**AMY-101**	**APT-070 mirococept**	**Eculizumab**	**ALN-CC5 cemdisiran**	**Coversin**	**Avacopan**
Classical pathway	x													
Lectin pathway	x	x												
Alternate pathway (factor B)				x		x								
Alternate pathway (factor D)			x		x		x							
C3								x	x	x				
C3a/3aR														
C5										x	x	x	x	
C5a/5aR														x
Company	Pharming behring shire	Omeros	Achillion	Novartis	Achillion	Novartis	RaPharma	Apellis	Amyndas	Inflazyme	Alexion	Alnylam	Akari	Chemocentryx

## Author Contributions

EC, AC, BC, AK, and JS contributed meaningfully and to the research and acquisition of data for this review, along with the subsequent analysis, drafting and revising of the paper. EC was the primary author due to his substantive drafting contributions, but all other authors provided equal contributions for the paper's creation.

### Conflict of Interest Statement

The authors declare that the research was conducted in the absence of any commercial or financial relationships that could be construed as a potential conflict of interest.
